# Adolescent peer struggles predict accelerated epigenetic aging in midlife

**DOI:** 10.1017/S0954579422000153

**Published:** 2022-04-05

**Authors:** Joseph P. Allen, Joshua S. Danoff, Meghan A. Costello, Emily L. Loeb, Alida A. Davis, Gabrielle L. Hunt, Simon G. Gregory, Stephanie N. Giamberardino, Jessica J. Connelly

**Affiliations:** 1 University of Virginia, Charlottesville, VA, USA; 2 Duke University, Durham, NC, USA

**Keywords:** adolescent, autonomy, epigenetic aging, friendships, longitudinal, peer, social relationships

## Abstract

This study examined struggles to establish autonomy and relatedness with peers in adolescence and early adulthood as predictors of advanced epigenetic aging assessed at age 30. Participants (*N* = 154; 67 male and 87 female) were observed repeatedly, along with close friends and romantic partners, from ages 13 through 29. Observed difficulty establishing close friendships characterized by mutual autonomy and relatedness from ages 13 to 18, an interview-assessed attachment state of mind lacking autonomy and valuing of attachment at 24, and self-reported difficulties in social integration across adolescence and adulthood were all linked to greater epigenetic age at 30, after accounting for chronological age, gender, race, and income. Analyses assessing the unique and combined effects of these factors, along with lifetime history of cigarette smoking, indicated that each of these factors, except for adult social integration, contributed uniquely to explaining epigenetic age acceleration. Results are interpreted as evidence that the adolescent preoccupation with peer relationships may be highly functional given the relevance of such relationships to long-term physical outcomes.

This study examined peer relationship difficulties as predictors of epigenetic aging. Identifying significant biomarkers of the aging process *prior* to the actual onset of disease is fundamental to establishing meaningful approaches to prevention of diseases of aging (Koss et al., 2021). Until recently, the long period of onset of classic aging symptoms has made such examination impractical in all but a few cases. Recent advances in epigenetic research, however, are now making it possible to assess markers of aging that can be tracked well prior to the onset of any actual disease. Early epigenetic aging algorithms – yielding measures of epigenetic age based on patterns of methylation within the epigenome – were found to yield estimates that correlated strongly with chronological age and added value in predicting future mortality (Horvath, 2013). By covarying out actual chronological age, these measures also produce an indicator of epigenetic age *acceleration.* This measure reflects the degree to which an individual’s epigenome suggests that they have already aged faster (or slower) than their chronological age would indicate (and is distinct from other indices that seek to capture not lifetime aging but rather the current pace of aging [Belsky et al., 2020]).

Further research, though, found that these early measures were often driven more by naturally unfolding biological processes than by external environmental factors (Palma-Gudiel et al., 2020). This in turn led to the development of second and third generation “epigenetic clocks” that were specifically designed to correlate with actual levels of physiological deterioration and also to be sensitive to environmental factors. The present study used a third generation epigenetic aging measure, DNAmGrimAge, which was designed to capture aspects of epigenetic aging that were most strongly correlated with actual indicators of physiological deterioration (Lu et al., 2019). DNAmGrimAge has been linked to a broad range of health indicators, including early mortality, time-to-heart disease, and time-to-cancer, and it has been found to outperform other existing epigenetic clocks (e.g., DNAmAge) when it comes to predicting many such health outcomes. Further, its link to mortality has been found to be robust across White, African American, and Hispanic individuals (Lu et al., 2019).

Although not yet well-studied, there is good reason to believe that epigenetic age acceleration will be linked to maladaptive social relationships earlier in life, in part due to the stress that relationship difficulties create. Social baseline theory, for example, suggests that the human brain is tuned to a default position of expecting to be in the presence of others who can act as potential support figures in times of threat or stress (Coan & Sbarra, 2015); the *absence* of supportive others is then viewed as decreasing the individual’s sense of relative safety. Theories regarding the role of social safety note the significant stress that occurs – and the adverse physiological effects that follow – when individuals perceive the social environment to be potentially threatening, for example in the face of interpersonal conflict (Brosschot et al., 2017; Slavich, 2020). Attachment theory has also long noted the importance of felt security in human functioning across the lifespan (Bowlby, 1980; Main et al., 1985), and variations in attachment have been associated with physiological risk factors such as altered immune function (Picardi et al., 2013) and cortisol responses (Pietromonaco et al., 2013).

Peer relationship struggles in adolescence seem likely to create the type of intense stress that could lead to loss of a sense of safety. Indeed, the combination of hormonal changes, neural development, and social demands in adolescence may make adolescent-era social stressors particularly powerful (Albert et al., 2013; Charles et al., 2001; Steinberg & Monahan, 2007).

One way to view and assess adolescent relationship stressors is in terms of the critical dialectical challenge that adolescents face: They must learn how to establish peer relationships that allow for autonomy and independence (including the ability to resist peer pressure) while still providing a strong sense of connection (Allen & Loeb, 2015). Mastering this challenge has been identified as a fundamental marker of social competence with long-term implications for adult relationships and functioning (Allen et al., 2020; Loeb et al., 2020; Oudekerk et al., 2014). Given the importance of establishing autonomy in adolescence, it is unsurprising that its absence in key relationships has been linked to higher levels of future hostile conflict (Allen et al., 1996) – precisely the kind of threat to social safety known to influence physical health outcomes. Similarly, failure to establish strong, connected relationships in adolescence seems likely to be inherently lonely and stressful, as well as indicative of difficulties that are likely to cascade forward to future social interactions, creating ongoing stress (Oudekerk et al., 2015). Adult attachment theory has found support for the idea that expectations regarding the availability of relationships that are autonomous yet connected can also become internalized (Main et al., 1985; Thompson, 2021). In sum, mastering this challenge in adolescence appears to reflect the essence of what it takes to become socially integrated and to establish social relationships as sources of safety versus as sources of conflict and threat.

Prior to and during adolescence, struggles establishing and maintaining positive connections with peers have been linked to higher levels of inflammation in adulthood (Allen et al., 2017; Copeland et al., 2014; Takizawa et al., 2015). Similarly, poor social integration in early adulthood has also been linked to markers of greater inflammation (Ford et al., 2019) while poor close friendship quality has been found to predict lower self-reported health quality in adulthood (Allen et al., 2015). In addition, over-involvement in romantic relationships in adolescence – a potential marker of autonomy difficulties – has in turn been linked to higher levels of adult blood pressure (Allen, Loeb et al., 2021). Whether and how these adolescent struggles with issues of autonomy and connection have been linked to more fundamental aging processes, however, has not been examined.

Evidence that chronic social stress is linked to long-term physical dysfunction suggests that social difficulties in adolescence are likely to predict such dysfunction (Fiorito et al., 2017; Hertzman, 1999; Miller et al., 2011). Exposure to stress has been found to potentially alter the epigenetic landscape across the lifespan, in part via its effects on the hypothalamic-pituitary axis and the glucocorticoid signaling system (Gassen et al., 2017). Stress exposure also appears linked to lasting changes in DNA methylation, forming a sort of “molecular scar” from this exposure (Zannas et al., 2015). Direct links between traumatic and threat-related experiences and accelerated epigenetic aging from a range of studies further support the existence of a stress-epigenetic aging connection (Brody, Miller et al., 2016; Brody, Yu et al., 2016; Jovanovic et al., 2017; Sumner et al., 2019). No research to date, however, has examined adolescent-era social relationship difficulties in relation to epigenetic aging processes.

This study assessed adolescent relationship difficulties via multiple methods. Self-reports of the degree to which an individual does or does not feel integrated into and motivated to participate in their social network are particularly useful for assessing the role of the individual’s perceptions of relationship quality. Direct observations of the extent to which close relationships are meeting the developmental need to establish autonomy and relatedness provide a more objective perspective. Finally, interview techniques can assess implicit internalized representations of relationships and allow for consideration of aspects expectations of relationships of which individuals may or may not be fully conscious. This study used the Adult Attachment Interview (AAI), which uses an individual’s description of early relationships to assess the individual’s *current* state of mind regarding close relationships. In particular, the AAI captures the degree to which an individual possesses, at present, an internalized sense of themselves as both autonomous and valuing of close relationships. This internalized sense – referred to as a “secure state of mind regarding attachment” (Main et al., 2002) – captures the fundamental sense of safety that comes with expectations of relationships as likely to be both autonomous and valuable.

A mediational “chains of risk” perspective suggests that early stressors, such as those that arise from struggles to establish autonomy and relatedness with peers, may predict future health outcomes by cascading forward to influence future relationship struggles both with peers and with romantic partners, which in turn have been robustly linked to poor health outcomes (Allen et al., 2020; Ben-Shlomo & Kuh, 2002; Holt-Lunstad et al., 2010; Oudekerk et al., 2015). Hence, this study considered adolescent-era relationship factors as potentially acting via similar mediating factors in adulthood. For example, we would expect potential links from difficulties in social integration in adolescence to difficulties in social integration in adulthood to accelerated aging, with similar pathways expected for observed autonomy and relatedness and for attachment states of mind. Alternatively, more direct, unmediated links may also exist, as struggles at a critical point in development, especially in the socially sensitive period of adolescence, could well have long-term effects on physiology (Blakemore & Mills, 2014). Cumulative lifetime stress has been previously shown to predict epigenetic aging (Zannas et al., 2015) and evidence of such socially linked weathering effects on health increasingly appear in the literature (Allen, Loeb et al., 2021; Allen, Loeb et al., 2018; Miller et al., 2011). Ultimately, both direct and mediated paths from relationship struggles to health outcomes appear plausible and warrant examination.

Several specific non-relational mediators were also considered given their historical links to health outcomes. A lifetime history of cigarette smoking was considered, given that one important limit to research on predictors of epigenetic aging is the possibility that markers of social stress are correlated with smoking behavior, which has been strongly related to aging (Joehanes et al., 2016; Norman et al., 2012). Smoking was primarily considered as a potential covariate, but the possibility that it would mediate the effects of social difficulties that predict lifetime cigarette use was also considered. Similarly, gender, racial/ethnic minority group membership, and adolescent family income were included as covariates in all analyses given their well-established links to markers of health and aging (Annandale, 2021; Khullar & Chokshi, 2018; Nguyen et al., 2014).

This seventeen-year, multi-method, prospective exploratory study utilized a diverse community sample to examine both direct and mediated pathways from adolescent struggles with processes of autonomy and relatedness to adult patterns of accelerated epigenetic aging. This study assessed struggles to establish autonomy and relatedness with peers from multiple vantage points, including self-reports and direct behavioral observations.

This study considered both pathways from relationship struggles to epigenetic age acceleration via four primary hypotheses:

Hypothesis 1: Observed struggles establishing autonomy and relatedness in relationships with close friends across adolescence will predict epigenetic age acceleration, with links potentially mediated by similar struggles with romantic partners in adulthood.

Hypothesis 2: An internalized lack of autonomy and valuing of relationships in adolescent states of mind regarding attachment will predict epigenetic age acceleration, with links potentially mediated by similar struggles in adulthood.

Hypothesis 3: Self-reported struggles in social integration with peers in adolescence will predict epigenetic age acceleration, with links potentially mediated by similar struggles in adulthood.

Hypothesis 4: Multiple social predictors of epigenetic age acceleration will contribute unique variance to epigenetic age acceleration after considering smoking history, but will also have some of their effects mediated via this history.

## Methods

### Participants

This report is drawn from a larger longitudinal investigation of adolescent social development in familial and peer contexts (Author, 2006). The final sample of participants (*N* = 154 [67 male and 87 female]) was a subset of the original sample of 184 adolescents first assessed at age 13 and for whom epigenetic data was able to be obtained at age 30 (*M* = 29.7, *SD* = 2.16). This reflected an 84% retention rate across the 17 years of the study. The final sample was racially/ethnically and socioeconomically diverse: 86 (56%) adolescents identified themselves as White, 48 (31%) as Black/African American, 2 (1%) as Asian, 1 (1%) as Hispanic, 1 (1%) as American Indian, and 14 (9%) as from other or mixed racial/ethnic groups. Adolescents’ parents reported a median family income at baseline in the $40,000–$59,999 range (*M* = $43,900, *SD* = $22,500).

Adolescents were initially recruited from the 7th and 8th grades of a public middle school drawing from suburban and urban populations in the Southeastern United States. Students were recruited via an initial mailing to all parents of students in the school along with follow-up contact efforts at school lunches. Families of adolescents who indicated they were interested in the study were contacted by telephone. Of all students eligible for participation, 63% agreed to participate either as target participants or as peers providing collateral information. All participants provided informed assent before each interview session, and parents provided informed consent. Interviews took place in private offices within a university academic building.

Participants were first assessed annually over a six-year period across adolescence from age 13.35 (*SD* = .64) to age 18.38 (*SD* = 1.04). For the adult follow-up self-report assessments, data were obtained from participants annually from ages 22.8 (*SD* = .96) to age 28.6 (*SD* = 1.02). In adolescence, participants also nominated the same-gender person they currently identified as “the peer to whom they were closest” to be included in the study. In adolescence, close peers came in during a visit along with the target participant and participated in observational assessments, as described below. Close friends in adolescence reported that they had known participants for an average of 4.3 to 5.7 years (*SD* = 3.1 to 3.8) across the various assessment periods. Participants could select a different person at each assessment, given that friendships change over time. Participants selected the same close friend approximately 40% of the time across any one-year period. Only 13% of participants selected the same close friend at age 18 as they had at age 13, indicating that participants’ interactions generally reflected a range of different relationships.

Romantic partner observations in adulthood were obtained for participants who were in a relationship for at least three months’ duration and in which the romantic partner was willing to come into our offices for an observational assessment. Romantic relationship assessments were obtained whenever a participant was in such a relationship and willing to participate at some point during three three-year windows. The result was that assessments were obtained at participant ages 23.8 (*SD* = 1.12), 27.4 (*SD* = 1.43), and 30.31 (*SD* = 1.24). Approximately 50% of participants had the same romantic partner from age 24 to 27, and 70% had the same partner from age 27 to 30.

### Procedure

In the initial introduction and throughout all sessions, confidentiality was assured to all study participants and adolescents were told that their parents would not be informed of any of the answers they provided. Informed assent was obtained from adolescents and informed consent was obtained from adolescents’ parents and from adult participants. Transportation and childcare were provided if necessary. Adolescent/adult participants, their parents, their peers, and their romantic partners were all paid for participation.

### Attrition analyses

Initial attrition analyses compared the 154 participants in the final sample to the 30 who were excluded because they lacked epigenetic data. Attrition analyses revealed no differences between these groups on any measures in the study. Among the participants with epigenetic data, analyses also compared the 113 who had romantic partner observational data to the 41 who did not. These analyses also revealed no differences between these groups on any measures in the study.

## Measures

### Epigenetic Age (Age 30).

The DNAmGrimAge measure was developed by combining DNA methylation markers of a range of physiological risk and stress factors. Unlike other epigenetic clocks, increased DNAmGrimAge does not directly indicate the number of years of increased aging, but instead captures and sums levels of methylation across a range of DNA sites that are indicative of increased mortality and morbidity risk (Lu et al., 2019). At the research arm of the local university medical center, trained technicians drew eight and a half milliliters of whole blood from participants into a PAXgene Blood DNA Tube (PreAnalytiX, Hombrechtikon, Switzerland). Samples were stored at −20°C for short-term storage (up to 3 months) then transferred to −80°C for long-term storage. DNA was extracted using the PAXgene Blood DNA kit (PreAnalytiX, Hombrechtikon, Switzerland) according to manufacturer instructions. DNA concentration was determined by Quant-iT™ PicoGreen® dsDNA reagent (Thermo Fisher Scientific, Waltham, MA, USA) per manufacturers’ instruction. Florescence was detected using a Tecan Infinite M200 Pro microplate reader (Tecan, Switzerland). 500 ng of DNA was bisulfite treated using a Zymo EZ DNA Methylation kit (Zymo Research, Irvine, CA) using polymerase chain reaction conditions for Illumina’s Infinium Methylation assay (95 °C for 30 s, 50 °C for 60 min×16 cycles). DNA methylation was assayed using the Illumina Infinium MethylationEPIC BeadChips. Briefly, a total of 4 μL of bisulfite converted DNA was hybridized to Illumina BeadChips using the manufacturer’s protocols. Samples were denatured and amplified overnight for 20 to 24 hr. Fragmentation, precipitation, and resuspension of the samples followed overnight incubation, before hybridization to EPIC BeadChips for 16 to 24 hr. BeadChips were then washed to remove any unhybridized DNA and labeled with nucleotides to extend the primers to the DNA sample. Following the Infinium HD Methylation protocol, the BeadChips were imaged using the Illumina iScan system (Illumina).

Raw.idat files were read and preprocessed using the *minfi* R package (Aryee et al., 2014; Fortin et al., 2017). The data set was preprocessed using noob for background subtraction and dye-bias normalization. All methylation values with detection *P* > 0.01 were set to missing (median sample: 669 probes, range: 255 to 4,026), and probes with >1% missing values (*n* = 5,788) were removed from further analysis. All samples were checked and confirmed to ensure that predicted sex matched reported sex. Additionally, samples were checked for excessive missing data (>5%) and unusual cell mixture estimates, which was estimated using the Houseman method as implemented in *minfi* (Houseman et al., 2012; Jaffe & Irizarry, 2014). All samples passed these quality controls. Principal components analysis, as implemented in the *shinyMethyl* R package, was used to examine batch effects (Fortin et al., 2014). The first seven principal components were examined using plots and potential batch effects were tested using linear models. Principal component 2, which accounted for 2.15% of the total variance, was associated with position on the array (*F*_(7, 245)_ = 14.93, *p* = 3.366e-16, adjusted *R*^2^ = 0.2789). Principal component 4, which accounted for 1.56% of the total variance, was associated with both bisulfite conversion plate and array number (bisulfite conversion plate: *F*_(2, 250)_ = 19.03, *p* = 2.307e-8, adjusted *R*^2^ = 0.1252; array number: *F*_(31, 221)_ = 7.98, *p* < 2.2e-16, adjusted *R*^2^ = 0.462). Bisulfite conversion plate and array number were associated with each other, as samples on the same array originated from the same bisulfite conversion plate. Because samples were randomized across plates and arrays, and proportions of variance explained by PC2 and PC4 were low, no batch correction method was used. The *ewastools* R package was used to assess Illumina quality control metrics and call genotypes and donor IDs to ensure the identity of repeated samples from the same individual (Heiss & Just, 2018). All samples passed Illumina quality controls.

To determine assay variability, we included one set of five technical replicates and an additional ten sets of two technical replicates. After quality control filters and normalization procedures were applied, the 5,000 CpGs with the most variable M values were used as input for calculating Pearson’s correlation coefficients among all pairwise combinations of samples. Pearson’s correlations of unrelated samples (different individuals) were below 0.8. Pearson’s correlations of technical replicates ranged from 0.987 to 0.996, indicating high agreement between technical replicates.

Unnormalized betas were filtered to include CpGs specified by Horvath as necessary for calculation of various clocks. The betas were uploaded to Horvath’s online DNA methylation age calculator (https://dnamage.genetics.ucla.edu), which provides measures of Horvath’s multi-tissue age estimator (Horvath, 2013), DNA methylation GrimAge (Lu et al., 2019), and cell type abundance. A sample annotation file was included. The options to normalize data and apply advanced analysis were selected. Technical replicates were used to determine measurement error of DNAmAge, the output of Horvath’s multi-tissue age estimator. The absolute difference of DNAmAge between technical replicate pairs was taken, as was the highest absolute difference in the set of five technical replicates. The median of the absolute difference was 2.02 years (range: 0.44–5.73 years), comparable to previous reports of measurement error being approximately 2.41 years (McEwen et al., 2018). Variability of DNAmAge in technical replicates did not differ by any demographic feature, including sex, race, or age.

### Attachment Interview and Q-sets (Ages 14 and 24) (George et al., 1996; Kobak et al., 1993).

This structured interview probes individuals’ descriptions of their childhood relationships with parents in both abstract terms, and with requests for specific supporting memories. Although the interview utilizes descriptions of past relationships, it does so to yield a marker of the individual’s *current* state of mind regarding themselves in close relationships. It yields an overall rating of security defined as a state of mind of being “autonomous yet valuing of attachment relationships.” For example, participants were asked to list five words describing their early childhood relationships with each parent, and then to describe specific episodes that reflected those words. Other questions focused upon specific instances of upset, separation, loss, trauma, and rejection. Finally, the interviewer asked participants to provide more integrative descriptions of changes in relationships with parents and the current state of those relationships. The interview consisted of 18 questions and lasted one hour on average. Slight adaptations to the adult version were made to make the questions more natural and easily understood for an adolescent population at age 14 (Ward & Carlson, 1995). These adaptations were not used for the adult interviews at age 24. Interviews were audiotaped and transcribed for coding.

The AAI Q-set (Kobak et al., 1993) was designed to closely parallel the Adult Attachment Interview Classification System (Main et al., 2002), but to yield continuous measures of qualities of attachment states of mind. Each rater read a transcript and provided a Q-sort description by assigning 100 items into nine categories ranging from most to least characteristic of the interview, using a forced distribution. All interviews were blindly rated by at least two raters with extensive training in both the Q-sort and with formal workshop training and certification for coding using the Adult Attachment Interview Classification System. Q-sorts were then compared with a dimensional prototype sort for *secure versus anxious interview strategies*, reflecting the overall degree of coherence of discourse, the integration of episodic and semantic attachment memories, and a clear objective valuing of attachment. The individual correlation of the 100 items of an individual’s Q-sort with a prototype sort for a maximally secure transcript was then used as that participant’s scale security score (ranging from −1.00 to 1.00). Inter-rater reliability, assessed via the intraclass correlation coefficient, for the final security scale score was .82 at age 14 and .71 at age 24, which is considered in the good to excellent range for this statistic (Cicchetti & Sparrow, 1981). Although this system was designed to yield continuous measures of qualities of attachment organization, rather than to replicate classifications from the Main et al. (2002) system, prior work has compared the scores obtained to a subsample (*N* = 76) of adolescent AAIs that were classified by an independent coder with well-established reliability in classifying AAIs. This was done by converting the Q-sort scales described above into classifications using an algorithm described by Kobak et al., (1993). Using this approach, an 84% match for security versus insecurity was obtained between the Q-sort method and the classification method (kappa = .68). Prior research in adolescent samples has also indicated that security assessed via this interview is relatively stable over a two-year period (i.e., *r* = .61) (Allen et al., 2004) and has expected relations to theoretically predictable outcomes including depression, aggression, and romantic behavior within adolescence (Chango et al., 2009; Miga et al., 2010; van Hoof et al., 2015).

### Autonomy & Relatedness in Close Peer Interactions (Ages 13–18).

Adolescent close peer dyads participated in an 8-min videotaped task during which they first answered questions about a hypothetical dilemma separately, and then were brought together to discuss their disagreement in a revealed differences paradigm (Strodtbeck, 1951). The topic of discussion was varied to be novel and developmentally appropriate across ages. For example, at age 13, participants and their close peers were instructed to imagine a situation in which twelve people with widely varying characteristics were stranded on Mars and only seven people would fit on the ship returning home. Adolescents and their peers first identified their seven chosen people separately, and then came together to discuss disagreements and make a final recommendation. At age 18, each member of the dyad was asked to select the top seven out of twelve people they would choose for a new reality television show they would be co-producing, and then came together to discuss their choices and create a final list. Using the Autonomy-Relatedness Coding Manual for Peer Interactions (Allen et al., 2001), researchers coded participants’ interaction style for behaviors promoting autonomy, defined as using reasoning and expressing confidence to advocate for their choices, and relatedness, defined as showing warmth and collaboration during the discussion. The approach of combining markers of autonomy and relatedness reflects the fundamentally interconnected nature of these two qualities and has been repeatedly empirically supported (Allen & Hauser, 1996; Allen & Loeb, 2015; Loeb et al., 2020). Overall scores for observed autonomy and relatedness were averaged across partners and across the six waves of the task as a marker of capacity to establish a dyadic relationship characterized by autonomy and relatedness. This dyadic perspective on observed interactions (i.e., considering both partners’ behaviors as part of the “dyadic dance”) has been repeatedly shown to be a reliable and valid means to capture capacity to establish healthy relationships (Allen, Porter, McFarland, McElhaney et al., 2007; Kansky, 2018). Autonomy and relatedness were coded reliably across raters with intraclass correlation coefficient values ranging from .63 to .87 across ages 13 to 18, all within the range of values considered to be good to excellent (Cicchetti & Sparrow, 1981). Although this measure is being used primarily as an inventory of interactions with different friends over time (vs. a scale with different items), the 6-year composite nonetheless displayed a moderately high degree of internal consistency (Cronbach’s α = .60).

### Autonomy and Relatedness in Romantic Partner Interactions (Ages 24, 28, 30).

Each adult participant-romantic partner dyad participated in an 8-min videotaped task in which they were asked to discuss an issue in their relationship that they had separately identified as an area of disagreement. The discussion began with target participants playing a recording they had made separately describing the problem and their perspective on it. Typical topics of discussion included money, jealousy, moving, friends, and career issues. Researchers then coded interactions using the Autonomy-Relatedness Coding Manual for Romantic Partner Dyads, a coding system derived from the peer autonomy and relatedness coding system described above (Allen, Porter, Mcfarland, Hare, et al., 2007). Specifically, participants’ interactions were coded for expressions of reasoning and confidence (i.e., autonomy) as well as warmth and collaboration (i.e., relatedness). As above, scores were averaged across partners and assessment to yield final scores for capacity to establish romantic relationships characterized by autonomy and relatedness. Interrated reliability ranged from good to excellent (e.g., intraclass correlation coefficients ranging from .69 to .90) across ages. When participants did not have a romantic partner, these data were considered missing and handled via the full information maximum likelihood procedures.

### Adolescent Social Integration (Ages 13–18).

Social integration in adolescence was assessed annually from age 13 to 18 via a six-item social integration scale created for this study. The scale used the format of Susan Harter’s Self-Perception Scale for Adolescents to reduce social desirability biases (Harter, 1988). Items focused on the extent to which participants perceived themselves to be integrated socially, for example, as “[setting] an example that other kids follow” and “[having] a lot of ideas that other kids listen to”; as well as the extent to which participants desired such integration, for example, “getting a lot of ideas about how to be from friends” and “[finding it important] that other teens like them.” Scores across the six waves were then summed and averaged to yield the final social integration score for adolescence. Internal consistency of aggregated scale scores was good across the six assessment waves (Cronbach’s α = .75). Construct validity for the scale is indicated by its modest links to the adolescent-era attachment instrument measuring autonomy and valuing of relationships (*r* = .24, *p* < .01) and to ratings by a close friend summed from ages 13 to 18 (*r* = .26, *p* < .001) of the degree to which the participant was viewed as socially accepted using the Social Acceptance scale from the Adolescent Self-Perception Profile modified to obtain ratings of one’s friend (vs. oneself, as in the original scale) (Harter, 1988).

### Adult Social Integration (Ages 23–29).

Social integration in adulthood was assessed annually from age 23 to 29 via the four-item social integration scale from the Social Provisions Scale (Cutrona & Russell, 1987). The scale includes items such as “I feel part of a group of people who share my attitudes and beliefs.” Scores across the seven waves were then summed and averaged to yield the final social integration score for adulthood. Internal consistency of aggregated scale scores was good across the seven assessment waves (Cronbach’s α = .89).

### Lifetime History of Cigarette Smoking (Multiple waves, ages 13–30).

Information regarding lifetime cigarette use was derived from two sets of data. First, level of smoking was assessed annually each year from age 13 to 18 in terms of number of packs smoked per day. At age 30, data was also obtained regarding year at which smoking began, year at which smoking ceased (if applicable) and average amount smoked in terms of number of packs per day. These two types of data were then harmonized such that where data were inconsistent, the higher level of reported smoking was used. The final score is calculated in terms of “pack years,” reflecting the product of the number of years smoked X the average number of packs of cigarettes smoked each year (e.g., if an adult participant reported smoking 1 pack/day for 2 years, but the adolescent data suggested more, than the adolescent data were used).

## Analytic plan

For all primary analyses, linear regressions were conducted using SAS PROC CALIS (version 9.4, SAS Institute, Cary, NC). To best address any potential biases due to attrition in longitudinal analyses, full information maximum likelihood methods were used with analyses including all variables that were linked to future missing data (i.e., where data were not missing completely at random). Because these procedures have been found to yield the least biased estimates when all available data are used for longitudinal analyses (vs. listwise deletion of missing data), the entire original sample of 184 adolescents was utilized for these analyses.

Analyses began by entering effects of blood cell counts, as these can be correlated with (and potentially confound) epigenetic age measures, and Lu et al. (2019) have found stronger predictions to physiological outcomes when accounting for this potential confound. In this analysis and all further analyses, the blood cell counts were estimated using the Horvath method for naïve CD8+ T cells, CD8+ CD28- CD45RA- T cells, plasmablasts (B cells) and naïve CD4+ T cells (Horvath & Levine, 2015). The Houseman method was used to estimate natural killer cells, monocytes, and granulocytes (Houseman et al., 2012). Chronological age was entered next, followed by participant demographic characteristics. By examining predictors of epigenetic age after accounting for blood cell counts and chronological age, the result is a prediction of epigenetic age acceleration (e.g., the extent to which a participant is epigenetically aging faster than their chronological age would indicate). Following entry of these variables, the primary predictor variables for each hypothesis were entered as described below.

## Results

### Preliminary analyses

Means and standard deviations for all variables used in the study are presented in Table 1. For descriptive purposes, intercorrelations among primary constructs are presented in Table 2.

We also examined possible moderating effects of gender, racial/ethnic minority group membership, and adolescent family income on the relation of social relationship qualities to epigenetic aging. Moderating effects were assessed by creating interaction terms based on the product of the centered main effect variables. No moderating effects were found for any of the analyses reported below.

### Primary analyses

Hypothesis 1. Observed struggles establishing autonomy and relatedness in interactions with a close friend in adolescence will predict epigenetic age acceleration, with findings potentially mediated by similar struggles with romantic partners in adulthood.

As shown in Table 3, when measures of autonomy and relatedness were added to the model following the relevant covariates, this step added significant variance to the overall model, with observed autonomy and relatedness in adolescence linked to epigenetic aging, such that lower levels of autonomy and relatedness in adolescence were linked to greater epigenetic age acceleration (*β* = .22, *p* < .001). Follow-up bootstrap mediational analyses, using the PROCESS macro in SAS (Hayes, 2019) indicated that the mediated pathway from autonomy and relatedness with close peers through autonomy and relatedness with romantic partners to epigenetic age acceleration was not significant (Indirect effect = −.025, 95% CI [−.095, .027]).

Hypothesis 2. An internalized lack of autonomy and valuing of relationships in adolescent states of mind regarding attachment will predict epigenetic age acceleration, with findings potentially mediated by similar struggles in adulthood.

Following the procedure described above, analyses next examined prediction of epigenetic age acceleration from attachment states of mind reflecting an internalized sense of autonomy and valuing of attachment at ages 14 and 24. As shown in Table 4, predictions were found from attachment states of mind at age 24, but not from these states of mind at age 14. Attachment states of mind indicative of both lack of autonomy and lack of valuing of attachment at 24 were predictive of greater epigenetic age acceleration at age 30 (*β* = −.24, *p* < .001). Follow-up bootstrap mediational analyses found evidence of a mediated pathway in which age 14 attachment states of mind predicted epigenetic age acceleration via age 24 attachment states of mind (Indirect effect = −.116, 95% CI [−.211, −.050]).

Hypothesis 3. Observed struggles in social integration with peers in adolescence will predict epigenetic age acceleration, with findings potentially mediated by similar struggles in adulthood.

Analyses next examined prediction of epigenetic age acceleration from self-reported social integration in adolescence and adulthood. As shown in Table 5, predictions were found from measures of social integration at both time periods, such that lower levels of social integration were predictive of greater epigenetic age acceleration (*β*’*s* = −.16 and −.12 from adolescence and adulthood respectively, *p*’*s* < .01 and .05). Follow-up bootstrap mediational analyses revealed no evidence of a mediated pathway from social integration in adolescence thru social integration in adulthood in predicting epigenetic age acceleration (Indirect effect = −.026, 95% CI [−.062, −.0002]).

Hypothesis 4. Identified social predictors of age acceleration identified above will contribute unique variance to epigenetic age acceleration after considering smoking history, but will have some of their effects mediated via this history.

**Figure 1.**
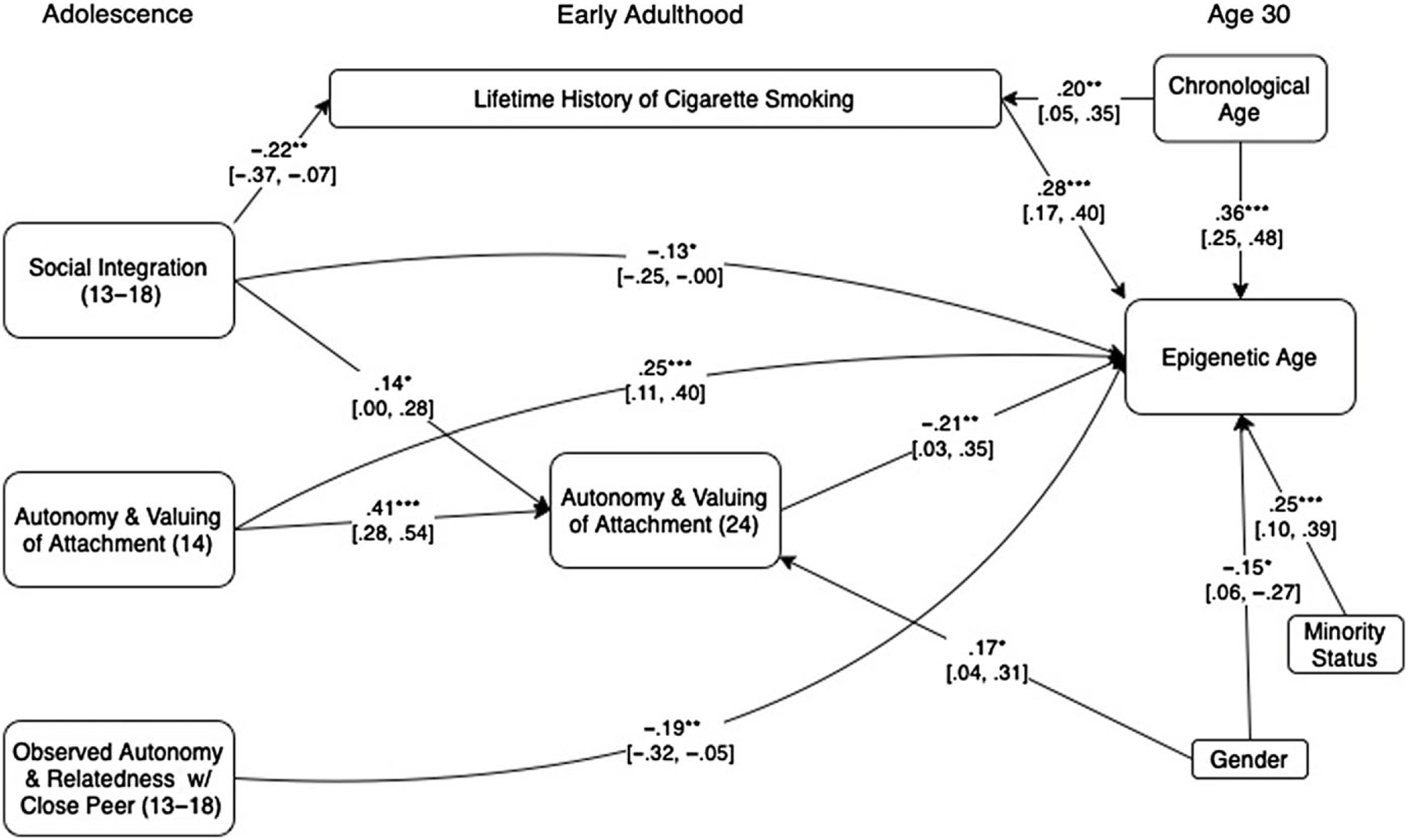
presents standardized estimates based on a path model in which all predictor variables were used to predict epigenetic age (residualized after accounting for effects of cell counts). Links across time between predictors were included where indicated via modification indices to improve model fit. For clarity, nonsignificant paths, variables that had no significant predictive or mediated relationships. and covariances among predictor variables are omitted. 95% confidence intervals are presented in brackets. ***p < .001. **p < .01. *p < .05.

We next used a path analytic approach to examine the potential predictors of epigenetic age acceleration simultaneously, along with a measure of lifetime cigarette smoking history to assess their unique, conjoint, and mediated predictions of epigenetic age acceleration. We began by entering potential mediated pathways from each adolescent-era predictor to aging via its corresponding marker in adulthood. Modification indices were then used to identify any additional links between predictor variables that would significantly improve model fit. Because intercorrelations among cell count measures led to problems with model convergence when included, these were instead handled by first regressing them out of the epigenetic age measure prior to employing path analyses. The result is the same in either case in terms of what is being predicted by substantive factors (i.e., epigenetic age after accounting for cell counts). The effect of cell counts thus do not appear in the final model in Figure 1, as they were taken into account at the prior stage.

The final model fit the data well (χ^2^ (12) = 12.41, *p* = .41, Goodness of Fit Index (GFI) = .99, Adjusted Goodness of Fit Index (AGFI) = .92, Root Mean Square Error of Approximation (RMSE) = .01) and accounted for 50.2% of the variance in epigenetic age as shown in Figure 1. For clarity, nonsignificant predictive links and covariances among predictors are omitted in the Figure. Unique predictions of epigenetic age acceleration were found from each of the previously identified predictors as well as from lifetime cigarette smoking history. Results of a regression model that also includes cell count data is presented in Table 6. These results indicated that the final block of five psychosocial predictors combined to predict 7.7% (*p* < .001) of the variance in epigenetic age after accounting for cell counts, demographic factors, chronological age, and lifetime history of cigarette smoking.

### Post hoc analyses

For comparative purposes, the model in Table 6 was also tested using the original Horvath measure of DNAmAge as the dependent variable. In this model, not shown, the block of 5 psychosocial predictors was significant (χ^2^ (5) = 12.35, *p* = .03), but only observed autonomy and relatedness with a close peer in adolescence was related to epigenetic age acceleration (*β* = −.19, *p* = .01).

## Discussion

The results of this study revealed a clear link between inability to establish social relationships characterized by autonomy and relatedness in adolescence and accelerations in epigenetic aging processes observable by age 30. Social relationship qualities were measured via direct observations, a coded interview, and self-reports, and evidence from each approach revealed links to epigenetic age acceleration. Further, analyses suggested that when considered jointly, the different markers appeared to capture at least somewhat unique aspects of relationship quality, in that several contributed uniquely to explaining epigenetic aging, even over and above a measure of lifetime cigarette use. When considered together, measures of struggles to establish social relationships characterized by autonomy and relatedness accounted for just under 8% of the variance in epigenetic age acceleration over and above variance accounted for by cigarette smoking and demographic factors. The combined variance accounted for by the relationship measures was comparable to the variance accounted for by lifetime history of cigarette smoking, providing an indicator of the substantial magnitude of the effects of observed.

Before considering the specific predictions observed, it is worth reflecting upon the potential mechanisms that can account for these broad findings. It should first be emphasized, however, that longitudinal predictions of this sort are *not* sufficient to support causal hypotheses. Further, given that epigenetic techniques have only recently been developed, we did not assess epigenetic aging at baseline data collection which additionally limits our ability to assess potential causal hypotheses.

Although causal relationships cannot be clearly established, these findings are highly consistent with the idea that social relationship difficulties can serve as both chronic and acute stressors and may act similarly to other stressors that have been linked to epigenetic aging (Brody, Miller, et al., 2016; Brody, Yu, et al., 2016; Jovanovic et al., 2017). Previously observed links between the glucocorticoid system and epigenetic changes appear as potentially viable routes of action for these linkages (Gassen et al., 2017), although research in this area is still in very early stages. At first glance, the stress created by maladaptive autonomy and connection processes with close others may seem quite distinct from the kinds of acute stress, typically involving significant trauma, previously linked to epigenetic aging. A developmental perspective can shed further light on these findings, however. Given the centrality of the developmental task of establishing autonomy and relatedness with peers in adolescence (Steinberg, 2019), struggles with this process may be particularly threatening during this period. Also, because adolescence is the first point in the lifespan in which truly adult-like relationships can begin to form, these relationships take on outsized importance as the first templates for future social relationships (Roisman et al., 2004). Further, while acute stressors may be more severe, relationship difficulties are often chronic, creating a continuous source of stress on the organism. Difficulties managing the balancing of autonomy and relatedness with peers have previously been linked to a range of problematic psychosocial outcomes, from depressive symptoms and long-term career difficulties, to ongoing difficulties with peer relationships more broadly (Chango et al., 2015; Henrich et al., 2001; Pakaslahti et al., 2002). Notably, both autonomy threats and connection threats in adolescence have now also been linked to indicators of deleterious physical changes well into adulthood (Allen et al., 2017; Loeb et al., 2021; Yang et al., 2016).

A link to epigenetic age acceleration was not found when assessing predictions from autonomy and relatedness in romantic interactions at ages 24 and 28. One possibility is that, beyond adolescence, autonomy and connection have already become well-established in most relationships; hence potential threats to autonomy and connection may come to feel less acute and intense. From this vantage point, adolescence may represent a particularly vulnerable period in the lifespan regarding issues of autonomy and relatedness. Given evidence that relationship stressors in adolescence can forecast adult physiological difficulties even if those stressors do not continue (Allen Loeb, et al., 2021; Allen, Loeb, et al., 2018; Miller et al., 2011), the possibility of such long-term weathering effects seems quite real. This also makes biological sense as aging effects are cumulative and we would expect that adult epigenetic age would be more influenced by events that happened in the past and have had time to exert their influence on aging. It is also quite possible, of course, that the lack of findings in adulthood simply reflects the smaller sample size of romantic partner observations or the overlapping variance between the adolescent and adult measures, particularly given that the adult measures were significantly related to epigenetic age in univariate correlations.

Findings regarding an internalized expectation of autonomy and connection in attachment states of mind were also linked to epigenetic aging, though with a different temporal pattern than observed displays of autonomy and relatedness. Attachment states of mind at 24 were predictive of epigenetic aging whereas those at 14 were not. From a developmental perspective, however, this pattern is actually more consistent with a perspective emphasizing the role of adolescent peer relationships than it might first appear. Attachment states of mind at 14 are believed to almost entirely reflect experiences within the family of origin (Booth-LaForce, 2014). Although there is considerable stability in these states of mind over time, relative decreases in an overall state of autonomy and valuing of attachment from 14 to 24 have been found to be strongly predicted by intervening *peer* difficulties (Allen, Grande et al., 2018). Hence, the temporal pattern of findings – with attachment states of mind becoming linked to epigenetic aging at some point between 14 and 24 – also points in the direction of adolescent peer experiences as a key long-term predictor of epigenetic aging. This may also be the reason for the apparent suppressor effect observed in final models in which attachment states of mind at 14 showed a positive link to aging (in contrast to the negative, though nonsignificant, link seen in simple correlations). Further supporting this post hoc interpretation, in the final path model, although not hypothesized, it was found that adolescent social integration was also predictive of a secure state of mind reflecting autonomy and valuing of attachment at 24.

Lower levels of self-perceived social integration were also related to accelerated epigenetic aging across both time periods. Although self-reports are problematic for many purposes, in this case, self-perception may be useful as a proxy for the likely stress felt by perceived absence of strong social support. Hence in accord with social baseline theory, it may be the *perceived* absence of membership in a group of supportive others that is most threatening (Coan & Sbarra, 2015).

Several limitations to these findings should also be kept in mind. In addition to the lack of baseline epigenetic data, the study also lacked information about stressors that may have occurred prior to adolescence and which may have affected both future social relationships and epigenetic aging. In particular, experiences of childhood maltreatment, which have previously been linked to epigenetic aging (Jovanovic et al., 2017), could easily have influenced future relationship development as well. Of course, it is also possible that prior childhood experiences had later effects that were observable precisely *because* they were mediated via intervening relationship experiences of the type measured in this study. Also, given that this study assessed a community sample in which rates of severe abuse experiences were relatively rare, this factor alone would seem unlikely to account for the magnitude and range of the effects observed.

A second limitation is that these findings were only partially replicated when using the original epigenetic clock measure (Horvath, 2013). This may be explained by the differences in the clocks. DNAmGrimAge estimates epigenetic age using DNA methylation estimators of serum protein levels of proteins which are markers of biological deterioration and which have been related to stress. Horvath’s clock estimates epigenetic age only on the basis of an algorithm which captures biological age and is less sensitive to environmental stressors. Although this is not surprising given that this original measure is considered less sensitive to environmental influences, it nonetheless adds an important cautionary note and indicates a clear need for future work to assess the replicability of these findings. The difference in findings between these two epigenetic clocks also makes clear the need for further research to explore just which epigenetic mechanisms are implicated in potential social stressor effects as well as how these mechanisms may translate into actual diseases of aging. Our lack of knowledge in this area should also make clear that no single measure is sufficient to quantify all aspects of biological aging (Hägg et al., 2019). A third limitation is that, given the composition of our sample, our examination of potential effects of genetic ancestry was, of necessity, somewhat crude, even though such ancestry differences have the potential to confound results (Philibert et al., 2020). However, given that the epigenetic aging measure used has been found robust across ancestry groups and that ancestry was considered as a covariate (and not found to be a significant moderator), this limitation is unlikely to have altered results. Finally, it should be noted that the final full model presented in Figure 1 is clearly exploratory in nature, including both hypothesized as well as non-hypothesized pathways between variables.

Given these limitations, this is nonetheless among the first studies to demonstrate long-term linkages between struggles in key aspects of social relationships, beginning as early as adolescence, and epigenetic aging. These linkages were observed from adolescence to epigenetic age acceleration at age 30, across multiple methods, and from a domain of social functioning previously identified as central to social development. Given previous findings on links from adolescent social experiences to other physical health outcomes, it is becoming increasingly apparent that the adolescent preoccupation with peer relationships, rather than being a quirk of this stage of the lifespan, may reflect a fundamental and biologically adaptive attunement to a domain with long-term consequences for health and for societal efforts to enhance health. For example, these findings may suggest new entry points for pediatricians assessing potential behaviorally-linked risks to future physical health beyond the usual focus on factors such as obesity, cigarette smoking, etc. Similarly, intervention approaches that directly target the quality of adolescents’ social relationships (Allen, Narr et al., 2021) might now warrant consideration not just for their immediate effects on adolescent well-being, but for their potential long-term implications for healthy aging. Finally, these findings suggest that parents trying to assess how their adolescent is faring might give greater weight to the quality of their ongoing peer relationships. Overall, then, these findings add growing urgency to calls to place a greater priority on processes of lifelong social connection and disconnection as precursors to key health outcomes (Holt-Lunstad et al., 2017).

## Figures and Tables

**Table 1.** Means and standard deviations of primary measures and demographic variables

	Mean	SD
Blood cell counts		
Naïve CD8+ T cells	285.8	44.3
CD8+ CD28- CD45 RA- T cells	3.28	3.13
Plasmablasts	1.94	0.191
Naïve CD4+ T cells	770.3	95.9
Natural killer cells	0.194	0.032
Monocytes	0.058	0.269
Granulocytes	0.634	0.104
Demographic factors		
Chronological age	29.7	2.176
DNAmGrimAge	37.6	4.95
DNAmAge	29.8	4.41
Lifetime cigarette use	2.49	4.17
Social relationship characteristics		
Observed autonomy & relatedness with close peer (Ages 13–18)	2.36	0.34
Observed autonomy & relatedness with Romantic partner (Ages 24, 27)	2.27	0.49
Autonomy & valuing of attachment (Age 14)	0.25	0.42
Autonomy & valuing of attachment (Age 24)	0.026	0.46
Adolescent social integration (Ages 13–18)	7.11	2.06
Adult social integration (Ages 23–29)	13.8	1.68

**Table 2.** Intercorrelations among primary constructs

	2.	3.	4.	5.	6.	7.	8.	9.
1. Epigenetic age (DNAmGrimAge) (30)	.39***	−.22**	−.24**	−.11	−.31***	−.23**	−.17*	.50***
2. Chronological age (30)	–	.14	.06	.09	.01	−.02	−.03	.20*
3. Observed autonomy & relatedness with close peer (13–18)		–	.39***	.30***	.20*	.06	.30***	−.01
4. Observed autonomy & relatedness with romantic partner (24, 27, 30)			–	.34***	.27**	.15	.22*	−.12
5. Autonomy & valuing of attachment (14)				–	.46***	.23**	.26***	−.10
6. Autonomy & valuing of attachment (24)					–	.23**	.27***	−.20*
7. Adolescent social integration (13–18)						–	.20**	−.22**
8. Adult social integration (23–29)							–	−.07
9. Lifetime cigarette use								–

*Note*. Participant age(s) at time of assessment are in parentheses.

*** *p* < .001.

** *p* < .01.

* *p* < .05.

**Table 3.** Predicting epigenetic age acceleration from observed behaviors displaying autonomy and relatedness

	β	DNAmGrimAge		
		95% C.I.	Δ*R*^*2*^	*R* ^*2*^
Step I. Blood cell counts				
Naïve CD8+ T cells	−.43****	[−.59, −.27]		
CD8+ CD28- CD45 RA- T cells	.18*	[.01, .33]		
Plasmablasts	−.21	[−.43, .01]		
Naïve CD4+ T cells	.40****	[.24, .56]		
Natural Killer cells	−.11	[−.27, .05]		
Monocytes	.04	[−.10, .18]		
Granulocytes	.38**	[.14, .62]		
Statistics for step			.25****	.25****
Step II. Chronological age				
Statistics for step	.45****	[.33,.57]	.17****	.42****
Step III. Demographic characteristics				
Gender (Male = 1, Female = 2)	−.20****	[−.36, −.08]		
Racial/Ethnic minority membership	.15*	[.01, .29]		
Family of origin income	−.04	[−.18, .10]		
Statistics for step			.10****	.52****
Step IV. Social relationship characteristics				
Observed autonomy & relatedness with close peer (Ages 13–18)	−.22****	[−.36, −.08]		
Observed autonomy & relatedness with romantic partner (Ages 24, 27)	−.09	[−.25, .07]		
Statistics for step			.05****	.57****

Note.

*** *p* < .001.

** *p* < .01.

* *p* < .05.

*β* weights are from final model.

**Table 4.** Predicting epigenetic age acceleration from autonomy & relatedness in attachment states of mind

	β	DNAmGrimAge		
		95% C.I.	Δ*R*^*2*^	*R* ^*2*^
Step I. Blood cell counts				
Naïve CD8+ T cells	−.46***	[−.61, −.30]		
CD8+ CD28- CD45 RA- T cells	.13	[−.02, .28]		
Plasmablasts	−.20	[−.42, .01]		
Naïve CD4+ T cells	.41***	[.24, .57]		
Natural Killer cells	−.08	[−.24, .07]		
Monocytes	.06	[−.07, .20]		
Granulocytes	.37**	[.13, .62]		
Statistics for step			.25***	.25***
Step II. Chronological age				
Statistics for step	.41***	[.30, .52]	.17***	.42***
Step III. Demographic characteristics				
Gender	−.14*	[−.26, −.01]		
Racial/Ethnic minority membership	.23**	[.09, .36]		
Family of origin income	−.09	[−.22, .04]		
Statistics for step			.10***	.52***
Step IV. Social relationship characteristics				
Autonomy & valuing of attachment (Age 14)	.09	[−.04, .23]		
Autonomy & valuing of attachment (Age 24)	−.24***	[−.37, −.11]		
Statistics for step			.04**	.56***

Note.

*** *p* < .001.

** *p* < .01.

* *p* < .05.

*β* weights are from final model.

**Table 5.** Predicting epigenetic age acceleration from self-reported social integration

	β	DNAmGrimAge		
		95% C.I.	Δ*R*^*2*^	*R* ^*2*^
Step I. Blood cell counts				
Naïve CD8+ T cells	−.45***	[−.60, −.29]		
CD8+ CD28- CD45 RA- T cells	.16*	[.02, .31]		
Plasmablasts	−.22*	[−.44, −.01]		
Naïve CD4+ T cells	.39***	[.22, .55]		
Natural killer cells	−.12	[−.27, .03]		
Monocytes	.01	[−.12, .13]		
Granulocytes	.36**	[.12, .60]		
Statistics for step	−.19**	[−.31, .07]		.25***
Step II. Chronological age				
Statistics for step	.41***	[.30, .52]	.17***	.42***
Step III. Demographic characteristics				
Gender	−.19**	[−.31, −.07]		
Racial/Ethnic minority membership	.25***	[.12, .38]		
Family of origin income	−.07	[−.14, .12]		
Statistics for step			.10***	.52***
Step IV. Social relationship characteristics				
Adolescent social integration (Ages 13–18)	−.16**	[−.27, −.05]		
Adult social integration (Ages 23–29)	−.12*	[−.23, −.01]		
Statistics for step			.04***	.56***

Note.

*** *p* < .001.

** *p* < .01.

* *p* < .05.

*β* weights are from final model

**Table 6.** Conjoint prediction of epigenetic age acceleration from relationship factors and lifetime cigarette use history

	β	DNAmGrimAge		
		95% C.I.	*ΔR* ^*2*^	*R* ^*2*^
Step I. Blood cell counts				
Naïve CD8+ T cells	−.35***	[−.49, −.20]		
CD8+ CD28- CD45 RA- T cells	.13	[−.00, .26]		
Plasmablasts	−.22*	[−.41, −.02]		
Naïve CD4+ T cells	.32***	[.17, .47]		
Natural killer cells	−.07	[−.21, .06]		
Monocytes	.08	[−.04, .19]		
Granulocytes	.37***	[.15, .58]		
Statistics for step			.25***	.25***
Step II. Chronological age				
Statistics for step	.36***	[.25, .47]	.17***	.42***
Step III. Demographic characteristics				
Gender	−.16**	[−.27, −.05]		
Racial/Ethnic minority membership	.24***	[.11, .36]		
Family of origin income	−.04	[−.16, .07]		
Statistics for step			.10***	.52***
Step IV. Lifetime cigarette use	.24***	[.13, .35]		
Statistics for step			.07***	.59***
Step IV. Social relationship characteristics				
Observed autonomy & relatedness with close peer (Ages 13–18)	−.18**	[−.29, −.07]		
Autonomy & valuing of attachment (Age 14)	.17*	[.05, .30]		
Autonomy & valuing of attachment (Age 24)	−.17**	[−.28, −.05]		
Adolescent social integration (Ages 13–18)	−.13*	[−.23, −.03]		
Adult social integration (Ages 23–29)	−.05	[−.15, .05]		
Statistics for step			.08***	.67***

Note.

*** *p* < .001.

** *p* < .01.

* *p* < .05.

*β* weights are from final model.
